# Cardiac findings in children with juvenile Dermatomyositis at disease presentation

**DOI:** 10.1186/s12969-017-0182-0

**Published:** 2017-07-11

**Authors:** Serdar Cantez, Gil J. Gross, Ian MacLusky, Brian M. Feldman

**Affiliations:** 1 Department of Pediatrics, Istanbul University, Istanbul Faculty of Medicine, Istanbul, Turkey; 2 Department of Pediatrics, University of Toronto, Division of Cardiology, The Hospital for Sick Children, Toronto, Canada; 3 Department of Pediatrics, University of Ottawa; Division of Respirology, the Childrens’ Hospital of Eastern Ontario, Ottawa, Canada; 4 Department of Pediatrics, Faculty of Medicine, and the Institute of Health Policy, Management & Evaluation, the Dalla Lana School of Public Health,, University of Toronto, Division of Rheumatology, The Hospital for Sick Children, Toronto, Canada

**Keywords:** Juvenile dermatomyositis, Heart disease, Screening

## Abstract

**Background:**

Juvenile Dermatomyositis (JDM) is a pediatric vasculopathy characterized primarily by skin and muscle involvement. Cardiac findings have been reported in children with JDM but have rarely been investigated in detail.

**Methods:**

We aimed to describe the relevant clinical and laboratory cardiac findings of a cohort of patients with JDM, followed at one centre, at disease diagnosis.

**Results:**

We performed a retrospective review of 105 patients with JDM, followed from 1991 to 2007. Six of 70 patients (9%, 6% of the entire cohort) had abnormal electrocardiographic (ECG) findings, while 26 of 54 patients (48%, 25% of the entire cohort) had abnormal echocardiographic (echo) findings. Many of these findings were either mild or unlikely to be a result of JDM.

**Conclusions:**

Our findings suggest that cardiac abnormalities at JDM disease onset are frequently seen, but are rarely significant findings due to disease; however, JDM patients should be considered for screening for cardiac disease as late cardiac complications are well recognized.

## Background

Juvenile Dermatomyositis (JDM) is characterized by a small vessel vasculopathy affecting children, characterized primarily by skin and muscle involvement. The etiology of JDM is not yet known [1]. Typical clinical findings of JDM include proximal muscle weakness, heliotrope rash, Gottron’s papules, nail fold capillary changes, skin calcifications and swallowing difficulties [2].

Cardiac abnormalities, sometimes serious, have been reported among the less common findings in JDM patients [2]. For example, Ravelli et al. have published long-term outcome and prognostic factors for JDM patients [3]. From Europe and Latin America 27 centers participated in this multicentre study, and 490 patients’ data was collected. They found that the cumulative frequency of damage to the cardiovascular system was 2.9% for 445 patients. Specific cardiac pathology and/or ECG and echo findings were not described in that study. Additionally, Pachman et al. reported a cohort of 21 patients with JDM. They found that 10 of 20 children had ECG abnormalities [4]. ECG changes were also examined in a study of 61 Korean juvenile and adult dermatomyositis patients [5]. In this study, ECG abnormalities were present in 6 of 14 JDM patients (37.5%); 3 patients had right bundle branch block and another 3 had ST changes. In a further study, Rider et al. aimed to validate the myositis damage index (MDI) [6]. In this article, they evaluated both adult polymyositis (PM) patients and JDM patients. Cardiovascular damage was more common in patients with PM than JDM; for the children, 2.8% had hypertension, and 0.7% had ventricular dysfunction.

ECG and echo changes have been recently investigated in a case-control study from Norway [7]. Sixty-one patients with an age and sex matched control group of healthy individuals were enrolled. This study aimed to compare cardiac findings of JDM patients with controls. Patients’ ECG and echo findings were investigated an average of 16.8 years after the disease diagnosis. In this study 7 patients developed pericarditis, 12 had hypertension, defined as systolic blood pressure > 140 mmHg, 10 patients had abnormal ECG findings (3 poor R-wave progression, 2 left ventricular hypertrophy, 2 right bundle branch block, one pathological Q-wave, one P pulmonale and one prolonged QTc). Using a similar design, the same group showed that heart rate variability is reduced in JDM patients (followed a median 13.5 years after disease diagnosis) [8], and that long-axis strain was reduced (a marker of systolic dysfunction on echo) in JDM patients followed a median of 16.8 years after diagnosis [9].

Cardiac disease may be serious in JDM and may be a cause of death. In a large series of North American children, heart disease contributed to mortality in 3 of 17 deaths in 329 patients. However, only 1 death (a child with myocarditis) appeared to be directly caused by cardiac disease [10].

Although the Norway studies are very important for demonstrating damage during follow-up, they do not give us information about cardiac findings at onset (the period when disease activity is often the highest). Early cardiac findings in JDM have rarely been investigated in detail; we therefore asked, what is the frequency of ECG and/or echo abnormalities seen at disease diagnosis in children with JDM?

## Methods

This study was approved by the Research Ethics Board at The Hospital for Sick Children, Toronto (SickKids). In our institution, every suspected JDM patient undergoes a detailed physical examination and a series of laboratory investigations including, for many, a cardiac work-up with electrocardiogram (ECG) and echocardiogram (echo).

We reviewed 105 patients newly diagnosed and followed at SickKids between 1991 and 2007. At each patient’s initial admission and/or first consultation visit, demographic data was noted, detailed physical examination including heart rate, blood pressure, skin examination, muscle weakness assessment and laboratory investigations were recorded, most often including a 12-lead ECG and standard transthoracic two-dimensional, M-mode, and Doppler echo assessment. All patients have been followed by a single pediatric rheumatologist, in a specialized JDM Clinic (BMF), and all have been treated according to our published standardized treatment plan [11].

For this retrospective study, demographic data and clinical findings, ECG and echo findings were obtained from clinical charts, rheumatology and cardiology clinic databases. All ECG and echo findings were reviewed by a single experienced pediatric cardiologist (GG). Heart rhythm, PR interval, corrected QT interval (QTc) and QRS duration were re-measured.

A standardized protocol had been used for all echocardiographic examinations [12]. For interpretation, we used institutional standardized z-scores, similar to those published [13], which our group has used in many publications. ECG standards followed previously published guidelines [14].

Descriptive statistics have been used to present our findings (proportions for categorical variables, means and medians with measures of spread as appropriate). Calculations were done with DataDesk 8.0.0 (Data Description, Inc., Ithaca, NY) and Microsoft Excel 15.35 (Microsoft, Redmond, WA).

## Results

105 patients (72 female) were included. Median age at the time of diagnosis was 6.9 years (range 2.0–17.6 years). Median time of follow up, at the time of our review, was 10.2 years (7 months – 15.8 years). (Table 1).

**Table 1:** Demographics

*N*	105 (72 girls)
Median (range of values) age at diagnosis, years	6.9 (2.0–17.6)
Median (range of values) follow-up time, years	10.2 (0.6–15.8)
Diagnosis	
JDM	102
Hypomyopathic JDM	1
Overlap myositis	1
Polymyositis	1
Number of Patients with ECG	69
Normal	63
Abnormal	6
Number of Patients with ECHO	52
Normal	26
Abnormal	26

Electrocardiograms were available for 69 patients. Thirty-six patients either did not have ECGs done, or the ECG was no longer retrievable.

Six patients had abnormal ECG findings (9%). QTc findings for all 69 patients were as follows: mean 416 ms, range of values 370–509 ms. QTc was abnormally prolonged in 5 patients. (Mean abnormal QTc was 462 ms.) Mean PR interval was 131 ms (94–214 ms). Mean QRS duration was 78.3 ms (56–112 ms). One patient had prolonged PR interval and wide QRS. (This patient also had moderate pulmonary insufficiency and mitral regurgitation by echo.)

Echo was available for 52 patients, of which 26 were normal (Table 2). Three patients had pericardial effusions; one of them was small and one was considered “trivial”. Isolated tricuspid regurgitation was seen in 3 patients. Six patients had tricuspid regurgitation combined with pulmonary insufficiency. One patient had both aortic and pulmonary insufficiency. Isolated pulmonary insufficiency was seen in 3, all considered trivial. One patient had mitral valve prolapse and mild mitral regurgitation and pulmonary insufficiency. Mitral regurgitation with pulmonary insufficiency was seen in one patient. One patient was found to have increased right ventricular end diastolic dimension. Another patient had decreased pulmonary vein D-wave velocity. Four patients had conditions that are almost certainly incidentally identified congenital abnormalities, and therefore unrelated to JDM: patent ductus arteriosus (PDA) with dilated left atrium and left ventricle, tubular hypoplasia of left aortic arch (THLAA), atrial septal defect, and ventricular septal defect. Ventricular function (left ventricular ejection fraction) was normal in all.

**Table 2:** ECG and ECHO Abnormalities

Abnormality	Number	Percent
ECG		
Normal	63	91.3
Prolonged QTc	5	7.2
Prolonged PR + wide QRS	1	1.4
ECHO		
Normal	26	50.0
THLAA	1	1.9
PER EFF	3	5.8
TR + PI	6	11.5
TR + PI + MR	1	1.9
TR	3	5.8
ASD	1	1.9
VSD	1	1.9
AI + PI	1	1.9
PDA + DIL LA + LV	1	1.9
PI	3	5.8
MVP + MR + PI	1	1.9
↑RVED dimension	1	1.9
↑ IVRT + TR + PI	1	1.9
PI + MR	1	1.9
↓ PV D wave vel.	1	1.9

The cardiac findings are listed along with the clinical features of JDM at diagnosis in Table 3.

**Table 3:** JDM measures sorted by cardiac abnormality

		Manual Muscle Testing (/10)^a^ [15]					Heliotrope rash			Gottron’s sign/papules			Other skin rash			Nail fold capillaries		
		n	median	IQR	min	max	n	Abnormal	%	n	Abnormal	%	n	Abnormal	%	n	Abnormal	%
ECHO	All (*n* = 105)	98	8	2	0	10	105	69	65.7	104	79	76.0	98	76	77.6	97	82	84.5
	Abnormal (*n* = 26)	26	8	3.75	0	10	26	19	73.1	26	21	80.8	25	19	76.0	24	21	87.5
	Normal (*n* = 26)	26	8	2.5	2	10	26	18	69.2	25	18	72.0	26	21	80.8	25	23	92.0
	Not done (*n* = 53)	46	8	1	2	10	53	32	60.4	53	40	75.5	47	36	76.6	48	38	79.2
ECG	All (*n* = 105)	98	8	2	0	10	105	69	65.7	104	79	76.0	98	76	77.6	97	82	84.5
	Normal (*n* = 63)	60	8	2.25	0	10	63	45	71.4	62	49	79.0	62	51	82.3	61	55	90.2
	Abnormal (*n* = 6)	6	8	1.5	5	10	6	4	66.7	6	6	100.0	6	5	83.3	6	5	83.3
	Not done (*n* = 36)	32	8	1.25	2	10	36	20	55.6	36	24	66.7	30	20	66.7	30	22	73.3

^a^As determined by the weakest muscle group at presentation using the 10-point Kendall scale

## Discussion

Our results suggest that many JDM patients have abnormal ECG and echo results at first presentation. Some of the findings, though, were mild and unlikely to be related to the underlying disease; for example, mild tricuspid regurgitation and/or pulmonary insufficiency are common and not necessarily abnormal. Although none of our patients presented with clinically serious cardiac dysfunction, and many of our patients had findings commonly seen in children without JDM, we believe that the high incidence of abnormalities is a reasonable justification for a cardiac work-up at disease onset. This, coupled with the reported accumulation over time of cardiac damage, suggests to us that careful cardiac monitoring over the disease course should be carried out in JDM patients [6, 7, 9].

We found more abnormalities at the outset of disease than other authors have seen over the course of the illness.^3^ This is likely because we used laboratory examinations to determine cardiac abnormalities; none of our patients were symptomatic, and none developed clinically meaningful cardiac disease during follow-up (although few had detailed laboratory examinations repeated over time as most were both asymptomatic and had normal heart rates and normal blood pressures for the duration of follow-up).

Our study should be interpreted in the light of possible limitations. Because our cohort goes back to the early 1990s, we have been unable to find some ECG and echo data; some patients did not have these investigations and others went missing. The implications of these missing data are unclear; however, as described above, none of these patients has gone on to have important cardiac morbidity.

## Conclusion

In conclusion, children with new onset JDM have a high frequency of cardiac abnormalities, mostly minor, as determined by ECG and echocardiography. We recommend that a cardiac workup should be considered in the initial workup for every suspected JDM patient.

## Abbreviations

ECG: Electrocardiogram
Echo: Echocardiogram
JDM: Juvenile dermatomyositis
MDI: Myositis damage index
PDA: Patent ductus arteriosus
PM: Polymyositis
QTc: Corrected QT interval
THLAA: Tubular hypoplasia of left aortic arch
